# Running with the Red Queen: the role of biotic conflicts in evolution

**DOI:** 10.1098/rspb.2014.1382

**Published:** 2014-12-22

**Authors:** Michael A. Brockhurst, Tracey Chapman, Kayla C. King, Judith E. Mank, Steve Paterson, Gregory D. D. Hurst

**Affiliations:** 1 Department of Biology, University of York, Wentworth Way, York YO10 5DD, UK; 2 School of Biological Sciences, University of East Anglia, Norwich Research Park, Norwich NR4 7TJ, UK; 3 Department of Zoology, University of Oxford, Oxford OX1 3PS, UK; 4 Department of Genetics, Evolution and Environment, University College London, London WC1E 6BT, UK; 5 Institute of Integrative Biology, University of Liverpool, Liverpool L69 7ZB, UK

**Keywords:** Red Queen hypothesis, coevolution, sexual selection

## Abstract

What are the causes of natural selection? Over 40 years ago, Van Valen proposed the Red Queen hypothesis, which emphasized the primacy of biotic conflict over abiotic forces in driving selection. Species must continually evolve to survive in the face of their evolving enemies, yet on average their fitness remains unchanged. We define three modes of Red Queen coevolution to unify both fluctuating and directional selection within the Red Queen framework. Empirical evidence from natural interspecific antagonisms provides support for each of these modes of coevolution and suggests that they often operate simultaneously. We argue that understanding the evolutionary forces associated with interspecific interactions requires incorporation of a community framework, in which new interactions occur frequently. During their early phases, these newly established interactions are likely to drive fast evolution of both parties. We further argue that a more complete synthesis of Red Queen forces requires incorporation of the evolutionary conflicts within species that arise from sexual reproduction. Reciprocally, taking the Red Queen's perspective advances our understanding of the evolution of these intraspecific conflicts.

## 1. Introduction

The Red Queen does not need changes in the physical environment, although she can accommodate them. Biotic forces provide the basis for a self-driving … perpetual motion of the effective environment and so of the evolution of the species affected by it. [1, p. 19]

Van Valen's ‘Red Queen hypothesis’ (RQH) emphasized the primacy of biotic interactions over abiotic forces in driving evolution. This was a revolutionary advance in biological thinking on the sources and modes of selection driving evolutionary change. Previously, the view of evolution by natural selection was that of a ‘hill climbing’ process, which shaped organisms to be well adapted to their environment. Because abiotic environments commonly change slowly with respect to the inhabiting organisms, evolution was thought to slow to a halt as the optimal phenotype is reached, recommencing only when conditions change. Biotic environments, by contrast, are themselves subject to evolution and so can change rapidly. According to the RQH, each adaptation by a species is matched by counteracting adaptations in another interacting species, such that perpetual evolutionary change is required for existence. Despite continued evolution, average relative fitness remains constant: evolution is a zero-sum game.

In the original paper, the RQH is proposed as a microevolutionary mechanism to explain a macroevolutionary observation: that the probability of taxon extinction appears independent of age. Van Valen named this the Law of Constant Extinction [1]. This law has proved controversial, and the strength of the supporting fossil evidence has been called into question [2]. Yet the broader insight that intrinsic biotic conflicts should drive perpetual evolutionary change, and that this could have macroevolutionary consequences, has been hugely influential [3,4]. Over the past 40 years, research inspired by the RQH has advanced our understanding of evolution in two major areas: first, the microevolutionary dynamics arising from biotic conflicts, and second, the role for biotic drivers in macroevolution. Citations of ‘a new evolutionary law’ reveal a recent surge of interest in the RQ, mirrored by recent increases in the numbers of published studies on the RQH (electronic supplementary material, figure S1). The RQ is therefore a pervasive concept in biology, but its usage has somewhat diverged as the RQ metaphor has been applied to different fields.

For many evolutionary biologists, the RQH is most strongly associated with debates surrounding the evolution of sex. The RQH provides a mechanism by which sexual species are protected from elimination by asexuals despite the latter's higher per capita reproductive rates. The maintenance of sexuals relies on rapid host–parasite coevolution such that parasites disproportionately infect common, asexual host genotypes, and rare genotypes, such as those possessed by sexuals, can avoid parasite adaptation. This body of theory [5] has been tested across a range of natural systems (e.g. [6–8]), providing compelling empirical support for this idea. However, Van Valen's original insight—that biotic conflicts are the primary driver of evolutionary change—has far wider implications. Here, we try to provide a holistic, biological view of the importance of RQ processes in interspecific conflicts, and how the study of the microevolutionary process described by the RQH has also been extended, beyond sex, to the maintenance of genetic diversity and rapid evolutionary change in communities. We then examine how intraspecific conflicts that follow from the evolution of sex can also be viewed in RQ terms.

## 2. Microevolution of interspecific conflicts

### (a) Which species interactions sustain perpetual evolution?

As originally conceived, the RQH encompassed all biotic conflicts over energy distribution (currency of the RQ) among species, thus unifying all trophic levels within the same framework [1,9]. As a result, Van Valen's RQH made no distinction between competitive and antagonistic (e.g. predator–prey, parasite–host) species interactions. Coevolutionary theory, however, suggests that these forms of biotic interaction vary in their propensity to sustain the perpetual, reciprocal coevolutionary cycles often called ‘Red Queen dynamics’. Competition is generally unlikely to drive perpetual evolutionary change of this kind. Coevolution of competitors tends towards character displacement [10], and thus weakens the intensity of the biotic interaction and the strength of selection over time. By contrast, RQ dynamics are more readily observed in models of antagonistic coevolution whereby the strength of selection acting on each species is roughly symmetrical. Symmetry is fulfilled in most host–parasite interactions, which have become the major focus of the microevolutionary research into the RQH, often in the context of the host–parasite coevolution selecting for sex [11,12]. The potential for RQ dynamics is expected to be limited when there is asymmetry in the strength of selection, such as that often found in many predator–prey interactions [13] (the ‘life-dinner principle’ [14]). However, important exceptions exist—in situations where prey have physical or chemical defences that make them dangerous to predators the strength of selection is likely to be more equitable [15].

### (b) Three modes of Red Queen dynamics

We define three broad classes of RQ dynamics distinguished by the modes of selection operating and the genetic architecture of coevolving traits (table 1):

(1) *Fluctuating Red Queen (FRQ)*, in which fluctuating selection drives allele frequency oscillations in both parties. For the FRQ to operate, interactions between antagonists require tight matching of traits under the control of few genetic loci. Exploiter populations track the common genotype of the victim species, and rare victim genotypes are at an advantage because they avoid exploitation. Allelic diversity is maintained within populations because matching pairs of antagonists' alleles undergo continuous time-lagged, negative frequency-dependent oscillations (e.g. [16]).
(2) *Escalatory Red Queen (ERQ)*, in which directional selection drives escalation of polygenic or quantitative trait values. The outcome of interactions is determined by the difference between antagonists' traits along a unidirectional axis [17]. Both antagonists are therefore under selection to ‘exceed’ the trait of the other species and coevolution proceeds as an arms race of recurrent selective sweeps. Arms races do not necessarily continue indefinitely and may either reach a stable equilibrium or drive one species extinct, bringing dynamic coevolution to an end [14]. However, RQ coevolutionary cycling can occur if the evolution of extreme trait values is bounded by costs or constraints and periods of escalation are followed by de-escalation [18].
(3) *Chase Red Queen (CRQ)*, in which local directional selection drives coevolutionary chases between exploiter and victim around phenotype space. Here, the coevolutionary game constantly changes. CRQ will generally occur when the interaction has a more complex genetic basis and hence can chase in multiple ways (in multidimensional phenotype space). Victims are under selection to increase phenotypic distance through *de novo* evolution of novelty, while exploiters are under selection to reduce phenotypic distance. Coevolution proceeds as a series of selective sweeps, which reduces genetic diversity within populations but drives divergence between populations. Sustained cycles of coevolutionary chase may occur through phenotype space whereby the direction and intensity of selection vary according to the relative locations of the species in phenotype space [19,20].

**Table 1.** Distinguishing the three modes of Red Queen. We define three distinct modes of RQ that are theoretically capable of sustaining perpetual coevolutionary cycling: Fluctuating Red Queen, Escalatory Red Queen and Chase Red Queen (defined in the main text). While we believe that each mode is necessary, it is less certain whether these modes are sufficient to encompass all manifestations of RQ dynamics in nature. It is possible (although given the intensive research over the past 40 years perhaps unlikely) that additional modes remain to be described theoretically.

	FRQ	ERQ	CRQ
genetic architecture	few major loci	polygenic or quantitative trait	polygenic or quantitative trait
basis of interaction	matching	difference	matching
selection mode	fluctuating	directional (unidimensional)	directional (multidimensional)
allele frequency dynamics	oscillations	selective sweeps	selective sweeps
adaptive landscape	multiple fitness optima	fixed fitness optimum	shifting fitness optimum

### (c) Red Queen over space and time

For FRQ coevolutionary interactions, the phase of allele frequency oscillations is likely to vary among populations. The genotypes or traits that are common and beneficial in one sympatric set of populations of interacting antagonists may be neither common nor beneficial in another. Thus, an antagonist species can be locally adapted to their sympatric interacting species population, but perform poorly in an interaction with an allopatric population. This has been widely demonstrated in host–parasite interactions, whereby parasites are better at infecting sympatric hosts, but allopatric hosts are better at resisting infection [21,22]. Consistent with this idea, parasites have been shown in natural systems to ‘track’ common host genotypes over time and subsequently drive down their frequency in the population [7,23,24]. Given that one genotype cannot dominate under this scenario, FRQ dynamics are predicted to maintain high levels of within-population genetic diversity (electronic supplementary material, box S1), and thus sexual reproduction (see above). Among populations, field collections of asexually reproducing invertebrates have revealed positive relationships between the diversity of clonal genotypes within a population and the frequency of infection by parasites [7].

**Figure 1.**
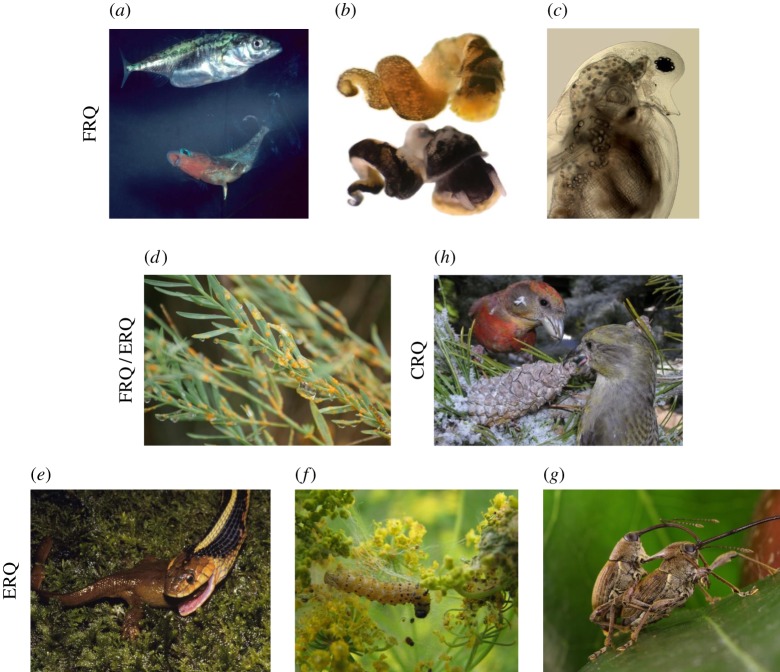
Natural systems used to explore Red Queen dynamics. (*a*–*c*) FRQ dynamics: (*a*) stickleback fish and trematode parasites, (*b*) *Potamopyrgus antipodarum* snails and trematode parasites, and (*c*) *Daphnia* waterfleas and microparasites. (*d*) Mixed FRQ/ERQ dynamics: *Linum marginale* and *Melampsora* rust fungus. (*e*–*g*) ERQ dynamics: (*e*) *Taricha* newts and *Thamnophis* snake predators, (*f*) wild parsnip and predatory webworms, and (*g*) *Camellia* and weevil predators. (*h*) CRQ dynamics: Crossbills and lodgepole pine trees. Photo credits: (*a*) M. Milinski; (*b*) C. Lively and G. Harp; (*c*) J. Wolinska and P. Juracka; (*d*) P. Thrall and J. Burdon; (*e*) B. Brodie III; (*f*) M. Berenbaum; (*g*) H. Toju; (*h*) C. Benkman. (Online version in colour.)

ERQ coevolution can give rise to spatial variation in the extent of coevolutionary escalation. Indeed, spatial variation is a potential signature of correlated defence and counter-defence trait evolution [25–28] (e.g. as between camellia pericarp thickness and camellia weevil rostrum length; figure 1). At the genomic level, bacteriophage phi-2 showed evidence of increased population divergence, as well as rapid evolutionary change, in response to ERQ coevolutionary dynamics with the bacterial host, *Pseudomonas fluorescens* [29]. Likewise, in CRQ interactions, divergence can be observed in the forms of the matching traits (e.g. [30,31]), such as the morphologies of lodgepole pine seed cones and the bills of seed predatory crossbills (electronic supplementary material, figure S2; cf. [32]). Moreover, there is evidence from a range of natural species interactions that is consistent with on-going selective sweeps driven by directional selection (e.g. [28,33–35]). The de-escalatory phase of ERQ dynamics is less well documented, although patterns consistent with ERQ cycles have been described for some of the defensive chemical and counterdefences in wild parsnip and its specialized webworm herbivore [28]. Contemporary phenotypic mismatches between levels of toxin and antitoxin in natural populations of newt versus its garter snake predator are also suggestive of a de-escalatory phase in a coevolutionary interaction [36].

### (d) Mixed modes of Red Queen

Recent empirical data suggest that the traditional theoretical dichotomy between fluctuating and directional reciprocal selection during coevolution may be an oversimplification. Mixed modes of reciprocal selection (i.e. combinations of fluctuating and directional selection) have been observed to operate within a given species interaction (i) at different loci within a genome, (ii) at different stages of a coevolutionary interaction, (iii) under different environmental conditions and (iv) at different spatial scales, as discussed below. This detailed view of the modes of selection operating has benefited from advances in experimental approaches to studying coevolution where the action of other sources of selection can be ruled out [37].

(1) *Different modes of coevolution within a genome.* Different modes of selection were observed operating at different loci within the same genome in nematode hosts experimentally coevolving with a bacterial parasite [38]. This suggests that infection/resistance is a multiphase process [39,40], and that different components of the immune response may be simultaneously under contrasting modes of selection. Patterns of genetic diversity across the genome are therefore probably shaped by a patchwork of evolutionary processes.
(2) *Change in the mode of coevolution over time.* Temporal changes in the mode of coevolution are also evident. As demonstrated in a recent experimental study of coevolution of the bacterium *P. fluorescens* and phage phi-2, a prolonged period of escalatory arms race coevolution can be a prelude to sustained FRQ dynamics [41]. An initial phase of escalating bacterial resistance and phage infectivity traits gave way to continual turnover of bacterial and phage genotypes with different specificities of resistance and infectivity, respectively, with no further change in the magnitude of these traits. This switch appeared to occur because accumulating costs of bacterial resistance progressively weakened the ability of bacteria to respond to directional selection [41].
(3) *Environmental impacts on the mode of coevolution.* Further observations of the *Pseudomonas*–phage experimental system suggest that the prevailing environment can shift the mode of coevolution, even in the early stages. While nutrient-rich liquid media supports an ERQ coevolutionary arms race, coevolution of the same bacteria–phage interaction in soil always follows a FRQ dynamic [42]. Once again this change in the mode of reciprocal selection appears to be mediated by the costs of bacterial resistance, which are elevated in nutrient-poor soil environments.
(4) *Different modes of coevolution across spatial scales.* Evidence from field studies of wild flax–flax rust populations reveals different modes of reciprocal selection depending upon the spatial scale of observations. At large spatial scales, covariation in population-level resistance and infectivity is consistent with ERQ coevolution [43], yet at smaller spatial scales, short-term within-population temporal change in resistance and infectivity traits and the underlying genes appears consistent with FRQ coevolution [44].

The discovery that mixed modes of reciprocal selection operate across a range of interspecific antagonisms broadens the scope for perpetual RQ coevolution, particularly in ERQ systems where arms races occur and de-escalatory phases have not yet been observed.

### (e) Red Queens in the community

The standard model of RQ interactions focuses on the ability of evolution to be sustained in pairwise interactions that themselves persist indefinitely. However, each pair of antagonistic species are probably only co-travellers for a finite period of time. The ‘end’ of interactions may be associated with mutual extinction (e.g. parasite removes its host species), with the victim evolving to remove the exploiter, or through the parasite fading out epidemiologically, because of evolved or externally forced changes in the demography/density of the host.

One property of antagonism is thus that any particular exploiter species is likely to be lost from a particular victim species. Pathogens able to attack the most common host species and impose selection (via FRQ dynamics) have been suggested to maintain species diversity in plant [45] and hybridizing communities [46] by preventing domination by one species. Clade selection for parasites that can shift to new host species may result, and exploiters that lose the ability to shift are doomed.

An important property of novel interactions is that they are likely to impose strong selection on both parties. The parasite finds itself in a novel host environment, in which rapid adaptation is likely, and the host is exposed to a novel parasite, which may interact with them via systems previously not exposed to selection. The early phase of novel interactions associated with host shifts is thus likely to be dominated by episodes of directional selection, rather than cycling of existing allelic variants. It is notable that granulysin, a gene with one of the strongest signatures of selection in the human lineage [47], is associated with resistance to *Mycobacterium tuberculosum*, a pathogen that emerged in humans following urbanization [48]. Thus, the ability of a particular interaction to create continued change may represent a fraction of evolution driven by current antagonistic partners. We need to expand our view of antagonistic interactions to the community context and recognize that an intrinsic property of antagonism is the presence of host shifts.

A further aspect of community context that requires consideration is extension of models beyond binary interactions. In nature, hosts carry a variety of antagonists, as well as related beneficial microbes. While some immune pathways may be specific to particular pathogens, others may have interplay with other pathogens and beneficial symbionts. Adaptation with respect to one party may thus impact upon others, such that the community context of antagonists and symbionts may modulate dynamics from that expected in simple binary interactions. For instance, the gut is host to pathogens, commensals and beneficial microbes. It has recently been observed that hybrid *Nasonia* fail to regulate the development of their gut microbiota, with hybrid larvae killed by the pathological impact of their own gut microbiome [49]. Here, selection on the innate immune system with respect to different microbiome members may in turn lead to divergence between species. Clearly, a community coevolutionary context will sometimes be essential to understand evolutionary patterns and outcomes.

## 3. Microevolution of intraspecific conflicts

When Van Valen conceived the RQ, he conceptualized it purely in terms of interactions between members of different species. However, our current, broader view—as continual evolution in the absence of environmental change—allows it to extend to intraspecific interactions. The evolution of sex, itself a potential consequence of interspecific RQ forces, establishes the possibility of conflicts over gene transmission during meiosis or reproduction, creating the conditions for the evolution of selfish genetic elements. It also establishes the possibility of conflict between the sexes, and between parents and offspring [50]. The RQ is a useful vehicle for exploring these interactions as (i) the interactions are antagonistic and (ii) the strength of selection is symmetric between interacting parties. We first define the major battlegrounds of conflicts—within genomes, between the sexes, within the sexes, and between parents and offspring—and then in each case assess the role of the RQ.

### (a) Red Queen and intragenomic conflict

Selfish genetic elements are genes (or sets of co-inherited genes) whose spread through populations imposes a cost to the individual that bears them [51]. For instance, a meiotic drive element in a heterozygote establishes overrepresentation of the chromosome bearing it in the gamete pool, commonly through preventing the formation of gametes that lack it. This behaviour aids the spread of the driver into the population, but does so at a cost to the individual carrier. Because drive elements are costly, mutations that prevent their deleterious action may spread in response to the presence of a driving chromosome, and these ‘suppressors’ produce selection on the drive element itself.

Intragenomic conflicts are known to undergo escalatory arms race dynamics, and are also likely to show FRQ behaviour. ERQ dynamics are best characterized in the *Drosophila simulans* ‘Winter’ meiotic driver, which comprises an X-linked gene *Dox* (*Distorter on X*) that drives against the Y chromosome in males [52]. *Dox* is suppressed by an autosomal gene, *Nmy* (*Not much yang*) [53]. Consistent with ERQ dynamics, there is evidence of recent selective sweeps at both loci [54], occurring more recently in both cases than the origin of the genes themselves, implying they are not in the first phase of an arms race, but an escalation.

FRQ behaviour has not yet been observed in nature for selfish genetic elements, but is predicted to occur both for meiotic drive [55,56] and cytoplasmic male sterility (CMS) [57]. In CMS, certain mitochondrial genotypes prevent pollen production in hermaphrodite plants. This phenotype diverts resource to ovules, which drives the maternally inherited mitotype into the population. CMS mitotypes select for the presence of restorer loci that rescue anther/pollen activity. There are genetic specificities in this system likely to support FRQ dynamics, with multiple CMS mitotypes alongside multiple restorers, with particular restorers effective against only certain mitotypes [58,59]. However, the frequent emergence of both CMS and meiotic drive in hybrid individuals suggests some CMS mitotypes and driving chromosome types become permanently suppressed within species [57]. Thus, RQ dynamics are limited in duration, and the continued existence of conflicts is associated with recurrent mutation to transmission distortion.

### (b) Red Queen and sexual conflict

Conflict between the sexes occurs because of differences in the evolutionary interests of sexes (in dioecious species) or of sex functions (in hermaphrodites) [60–62]. It reflects sex differences in costs of reproduction and situations in which the genes residing in each sex, or sex function, can gain fitness by causing the other sex to invest more [60,62–65]. For example, if males gain fitness through investing in longer matings, but females simultaneously lose fitness because long copulation is costly (e.g. predation risk), there will be sexual conflict over mating duration [66]. This can lead to sexually antagonistic selection [65,67,68].

Sexual conflict is most intense when current mates have low interest in the success of their partner's future reproductive bouts; for example, where there is promiscuity and low relatedness between mating partners [69]. The interactions show equality or symmetry—*both* parties have to interact to gain fitness, unlike the asymmetrical relations between predators and prey. However, intersexual interactions can develop asymmetry as it nearly always pays for males, but not necessarily for females, to mate [60].

Surprisingly, despite the power of the RQ metaphor and its potential to illuminate sexually antagonistic interactions, it has seeped into the study of sexual conflict rather than being a central part of its development [67]. The application of the RQ in sexual conflict has generally been rather vague, partly because the RQ has never been clearly defined for sexual conflict, and also because of conceptual confusion more generally about what forms of dynamic evolutionary change are defined by the RQ.

To understand the explanatory power of the RQ in sexual conflict we can consider how applicable it is to either of the major routes by which sexual conflict may be manifested. Sexual conflict is commonly partitioned according to genetic architecture [67]. *Intralocus sexual conflict* can occur if (i) alternative alleles of the same gene have differential effects on male versus female fitness, or (ii) the expression of a single allele has a different optimum level in males versus females—and hence cannot simultaneously be optimized for both sexes. We expect the potential for RQ dynamics to be limited under intralocus sexual conflict because the underlying alleles involved are not free to cycle through time and space.

In *interlocus sexual conflict*, the two sexes express different genes that influence a single shared trait (e.g. the different gene(s) in males and females that affect mating frequency) [60,65]. There is abundant experimental evidence of traits that function to increase male fitness at the expense of that of their mates, and of counterselection to minimize costs that these traits impose on females [65,70–72]. We envisage that a core feature of interactions between males and females is the coordination of a complex series of events in courtship and mating required for successful reproduction. An efficient way to initiate this is for females to use cues (such as the receipt of seminal fluid molecules) from courting or mating males to initiate reproductive processes such as oviposition/egg production only once a mating has occurred. However, it then becomes possible for males to evolve to highjack or exploit those pathways to cause females to invest more than is optimal from the female point of view. For instance, sex peptide (a seminal fluid protein) reduces the likelihood of female remating in *Drosophila melanogaster*. The origin of the sex peptide receptor pre-dates the evolution of sex peptide itself [73]. Sex peptide appears to have hijacked the receptor's ancestral function. This is akin to sensory exploitation as envisaged under sexual selection [74] and creates a two-locus sexual conflict system.

A suite of dynamic interactions are possible under interlocus conflict [60,75–79]. While some of these involve evolution to equilibrium, and in others only one sex is expected to evolve, three are characteristic of the RQ (see electronic supplementary material, box S2 for discussion of the evidence from sexual conflict in support of FRQ, ERQ and CRQ). The type of dynamic expected to occur under interlocus sexual conflict depends on mechanistic details such as dominance and the number of loci involved [60,79]. This parallels thinking about the importance of the extent of gene-for-gene models versus other mechanisms in interactions between hosts and parasites. Improved understanding of the evolutionary dynamics of sexual conflict clearly requires a deeper understanding of its mechanistic underpinnings.

Theory suggests two other features of RQ dynamics under sexual conflict. First, if traits under sexually antagonistic coevolution are also subject to other components of natural selection then the likelihood of RQ dynamics will be reduced [79]. This is consistent with RQ theory, which stresses the importance of low pleiotropy in interacting traits [80]. Second, more than one mode of RQ dynamics may operate simultaneously, and, as observed from host–parasite interactions, different modes may also operate through time from the origin to maintenance of a sexually antagonistic interaction [60,81].

Conflict within sexes arising from sexual selection can also represent a potent opportunity for Red Queen dynamics. Intrasexual asymmetric competition displays ERQ dynamics in a number of cases, as evidenced by highly elaborate traits. Sperm competition associated with polyandry can drive the evolution of extreme ejaculate sizes, and variation in sperm morphology and size [82]. FRQ dynamics are also evidenced in the evolution of alternate mating tactics, such as calling and satellite male crickets [83]. Some of these traits show frequency-dependent cycling. For example, the three male mating types of the side-blotched lizard (*Uta stansburiana*) cycle in frequency, owing to a non-transitive (i.e. ‘rock–paper–scissors’) interaction [84].

A requirement for the RQ is that there is sufficient continued genetic variation fuelling ongoing sexual conflict. The continued running of the RQ is supported by genomic evidence. From sea urchins [85] to *Drosophila* [86,87], it is clear that genes involved both explicitly and more peripherally in reproduction often show rapid rates of evolution, often owing to positive selection [88–90]. However, it should be cautioned that only a fraction of these changes will be due to RQ processes associated with sexual conflict. Sex-biased genes are typically identified using whole-transcriptome profiling, and this approach amalgamates several types of genes into one class. For example, only some sex-biased genes have a direct role in gamete production or fertilization, while many others are related to other sexual dimorphisms [91]. The uncertain role of the RQ is also a result of doubt over what proportion of these genes is involved in interlocus conflict versus how many of them simply reflect intralocus conflict over optimal expression between males and females, or are a product of sexual selection on one sex only. This mishmash of different types of genes into studies of sex-biased genes results from the fact that genome-wide expression and sequence data are relatively cheap and easy to generate compared with detailed tests of interactions, functionality and fitness effects. To demonstrate a role for the RQ conclusively, studies integrating functional genetics and sex-specific fitness or phenotypic effects (e.g. [92]) are required.

### (c) Red Queen and parent–offspring conflict

Trivers [93] recognized that where an individual reproduces sexually, parental and offspring optima for resourcing diverge whenever care has a cost to the parent. Divergence in parental and offspring optima are a potential source of evolutionary conflict where offspring can manipulate parental investment. The divergence in the interests of individual offspring and their resourcing parent is greatest where a parent changes sexual partner. Polyandry both decreases relatedness of the current sibling to future (half-) siblings (widening the gap between parental and offspring optima for investment) and produces sexual conflict over resourcing, as their partner's future offspring will be unrelated [94]. Candidate genes involved in sexual conflict over resourcing can be ascertained from transcriptome profiling, which allows genes with parent-of-origin expression to be identified. The unusual expression pattern of these genes is thought on some occasions to be the result of interlocus sexual conflict between the mother and father over resource allocation, played out through the developing fetus [94], such as in the classic example of human insulin-like growth factor 2 and its receptor. These loci have been through countless rounds of adaptation and counter-adaptation (ERQ), and mis-expression has severe phenotypic consequences for offspring [95].

It has been suggested that vivipary provides the most probable ground for the operation of RQ within parent–offspring interactions. Crespi & Semeniuk [96] argued that placentation in mammals created extended and more intimate parent–offspring interactions, thus intensifying conflicts. Provisioning of seed in plants, orchestrated by both maternal plant and seed genotype, is likewise a potent potential battleground [97]. As expected from a conflict model, large seed sizes (typical of over-exploitation of maternal plant by the seed) are disproportionately observed where pollen comes from another population, so long as this population is not selfing (which would reduce conflict) [98]. Furthermore, genomic data are consistent with RQ dynamics. Mammalian genes that show parent-of-origin differences in expression evolve more rapidly [99–101]. However, aside from the classic example of human insulin-like growth factor 2, it is unclear what fraction of imprinted genes is associated with parent–offspring conflicts. Similar to the potential role of the RQ in driving accelerated evolution of sex-biased genes, detailed gene-by-gene studies of the interactions and functions of each gene identified in transcriptome profiling are required to quantify the importance of the RQ in the evolution of these genes.

## 4. Conclusions and prospects

For the past 40 years, Van Valen's RQH [1] has transformed our understanding of how biotic interactions can shape the evolution and diversity of species in nature. The RQH continues to stimulate research on interspecific antagonistic coevolution, notably host–parasite coevolution. The applicability of the concept has even spilled over into medicine (electronic supplementary material, box S3), whereby understanding the relationship between the adaptive immune system and disease evolution may aid in the treatment of infection and symptoms.

The RQ is probably a more dominant driver of evolutionary change in nature than is presently recognized. Additional systems should now be used to test for the role of RQ coevolution in maintaining trait variation and the ubiquity of sex. Furthermore, the genomic revolution has afforded researchers an unprecedented, detailed and unbiased view of the RQ's role in shaping adaptation at the molecular level. Recapitulating phenotypic patterns at the molecular level has revealed that the RQ maintains high levels of within-population genetic diversity (electronic supplementary material, box S1), imposes multiple modes of selection on the genome and can drive rapid evolutionary change. Development of the functional genetics of interactions (within and between species) and comparative analyses has also revealed that ‘fast-evolving genes’ are commonly those at the interface of biotic interactions. Exploring patterns of molecular coevolution may serve to further uncover the signature of the RQ.

Finally, the adoption of a broad definition of RQ dynamics will offer a wider scope for the investigation of perpetual coevolution. For example, the previous lack of application to intraspecific conflicts may have been owing to conceptual uncertainty about RQ dynamic evolutionary changes. There are numerous parallels between inter- and intraspecific coevolutionary dynamics: the RQH may provide a new evolutionary framework for studying intraspecific conflicts, which may often be better described by ERQ and CRQ dynamics. In addition, future work may also explore interspecific RQ coevolution with more ecological realism. Virtually all organisms live in diverse communities where any interaction has more than two players, and the evolution occurs within a network. If antagonists can switch to new victims in the community and victims can be attacked by multiple enemies, evolutionary changes may occur via ERQ, CRQ or mixed modes of selection, indefinitely.

## Supplementary Material

Supplementary boxes

## Supplementary Material

Figure S1

## Supplementary Material

Figure S2

## Supplementary Material

Legends for Supplementary Figures
