# Supplementary material for: Running with the Red Queen: the role of biotic conflicts in evolution
Source: Proc Biol Sci. 2014 Dec 22;281(1797):20141382. doi: 10.1098/rspb.2014.1382 (PMC4240979; doi:10.1098/rspb.2014.1382)
Supplement: Supplementary boxes [file rspb20141382supp1.docx]

**Box S1. Red Queen dynamics and genetic diversity**

Consider two populations or species, which were once interbreeding but are now separate. In Fig. S2a, RQ dynamics are manifested as allele frequency oscillations driven by negative, frequency-dependent selection (see definition of FRQ in main text). These dynamic cycles continue indefinitely, maintaining within population genetic diversity over long periods of time and through population splits or speciation events. The genetic basis for FRQ is assumed to be a matching allele (MA) model, whereby a single locus with two or more alleles in each of the antagonists has matching specificities – a lock and key – that allow each antagonist to exploit/resist the other. In host-parasite interactions, whereby parasite’s possessing a certain allele must exactly match that of the host to evade the immune system and successfully infect [1], the evidence for such tight specificity is growing [2-5]. In Fig. S2b, RQ dynamics are characterised by successive waves of mutation in each antagonist, either as a result of an escalatory arms race or a coevolutionary chase (see the definitions of ERQ and CRQ, respectively, in the main text). A novel mutation appears that confers an advantage for one antagonist against the other (e.g. a resistance mutation in a host). It is then the turn of the antagonist to evolve a countermeasure and so on, locking both antagonists into a continuing arms race. These mutations are generally assumed to occur at different loci – a gene for gene (GFG) model – which finds good empirical support in plant-pathogen interactions [6]. The result is a succession of sweeps of advantageous alleles through a population, where any genetic diversity within a population is transitory. Following a population split, each daughter population follows its own evolutionary trajectory and diverges inexorably from the other and from the ancestral population.

Different forms of RQ dynamics predict different patterns of within population genetic diversity and between population/species genetic divergence. Under FRQ, higher levels of within population diversity (and diversity-generating strategies, such as sex) may be maintained by selection pressure from coevolving antagonists. Genes subject to FRQ are expected to exhibit high levels of diversity within populations and the preservation of ancient polymorphism among species. Genes of the major histocompatibility complex (MHC) are cited as a classic example [7]. These genes are phenomenally polymorphic, with hundreds of alleles that may differ in their specificity to present different parasite antigens to the vertebrate immune system. Allelic lineages can be maintained for very long periods of time. For example, given a pair of alleles present at an MHC locus in humans, one of these may be more similar to an allele found in a chimpanzee than to the other human allele [8, 9]. More recently, genome scans, examining polymorphism in humans and chimpanzees, have uncovered a number of loci, in addition to the MHC, that also show this pattern [10]. Many of these are non-coding DNA but appear to be located near to genes involved in immunity. These data (particularly for the MHC) are often used in support of FRQ, but it is important to note that these patterns are also consistent with heterozygote advantage, where for example, MHC heterozygotes are protected against a wider range of pathogens than homozygotes [7]. Although polymorphism is maintained by the antagonistic interaction, there is no coevolution between antagonists. Providing a clear picture of whether negative frequency-dependent selection or heterozygote advantage is the main driver of diversity at the MHC or other loci has proved elusive.

Because ERQ and CRQ generate directional selection they are predicted to increase genetic divergence between related species (or isolated populations of a given species) as each accumulates a different succession of mutations that, temporarily, give it an advantage against its antagonist. For any genes that are the target of repeated rounds of selection, one might expect the rate of amino acid substitution to be elevated relative to neutral predictions. (A number of methods have been developed to test this relationship, and which generally utilise ratios of non-synonymous to synonymous substitution to compare rates of adaptive to neutral evolution [11]). The selective sweeps characteristic of ERQ and CRQ also leave a signature of reduced polymorphism around genes under selection. Thus, an adaptive mutation will quickly rise in frequency to become fixed during a selective sweep and drag with it neutral polymorphisms that happen to be immediately adjacent to it when the mutation first arose (genetic hitch-hiking). Genomic signatures of the RQ are found in studies of genetic diversity within and among *Drosophila* species [12, 13]. Genome-wide analyses reveal higher rates of adaptive evolution in immune genes than in the rest of the *Drosophila* genome, but with selection focussed on specific immune genes, notably RNAi genes that protect against viral infection. Interestingly, analysis of populations of Sigma virus in *Drosophila* indicate recent selective sweeps, suggesting persistent arms races between host and parasite populations [14].

**Box 1. Sexual conflict dynamics and the Red Queen**

**1. FRQ.** Evidence for FRQ in sexual conflict would be tight tracking of allele changes at one or few loci subject to sexually antagonistic selection in males and females over time, together with evidence of frequency-dependent fitness. Consistent with this scenario is the interaction between gametes in marine invertebrate broadcast spawners [15, 16]. Conflict is between the increasing the rate of fertilisation versus the need to avoid polyspermy [17]. The interaction between sperm and egg seems to satisfy allele matching. For example, the efficiency of the penetration of the sperm through the vitelline membrane is significantly compromised in crosses from different populations/species (e.g. [18, 19]). Evidence for coevolution at the sequence level between the cognate partners has now also been shown [16].

**2. ERQ.** The signature of ERQ is directional selection and escalation along a single phenotypic axis. An example is the interaction between grasping and antigrasping adaptations in pondskaters and diving beetles [20, 21]. Here, the sexual conflict over mating frequency has selected ever-more efficient grasping structures in males and antigrasping adaptations in females. In pondskaters, this appears to reach a stable equilibrium, but recurrent escalations and deescalations are possible. Escalation is a general pattern in sexually selected reproductive characters and ERQ may be a hallmark of intense intrasexual selection. Where this intersects with sexual conflict is that intrasexual contests may often intensify the interactions with the other sex.

**3. CRQ.** When sexual conflicts are mediated via interactions between multiple loci, coevolution along multiple axes of phenotypic space is possible. Instead of exaggerated phenotypes one expects evidence of evolution spinning off along multiple directions, and hence frequent incompatibilities between the sexes across space and / or time [22]. Between population crosses have been used to detect the footprint of sexually antagonistic coevolution and frequently detect significant genotype-by-genotype interaction [23]. However, exclusive predictions consistent with sexual conflict are hard to derive [24]. Sexual conflict may often select for co-option by one sex of naturally selected traits and systems in the other, which could then lead to CRQ dynamics.

**Box S3. The Red Queen in the clinic**

A major contribution of the RQ for health and medicine is for understanding virus evolution. Here, the biotic environment of the virus is its host’s immune system [25]. Although this is not the RQ in the strictest sense, since changes in the host are plastic rather than heritable, the rapid response of acquired immunity to recognise and neutralise virus antigens drives the virus to evolve new epitopes able to escape host immunity. For HIV, the persistence of infection within a host is achieved in large part by the ability of HIV to mutate into new variants, particularly in a hypervariable region of its *env* protein, able to evade the current repertoire of host antibodies or cytotoxic T lymphocytes [26]. For influenza, new viral variants, generated by either antigenic drift or antigenic shift, facilitate new rounds of transmission within host populations that have immunity to older strains [27]. These, and other viruses, appear hard-wired to run with the RQ, encoding hypermutable sites in proteins exposed to the immune system or easily recombined genomes that give high mutational capacity. Similarly, *Trypanosoma brucei*, a protozoan parasite of humans and livestock, encodes hundreds of variable surface glycoproteins (VSGs), only one of which is expressed by a given parasite [28]. The expression of VSGs is switched at a low rate, leading to FRQ dynamics of successive parasite VSGs dominating within an infection, each to be neutralised by an immune response and replaced by another VSG. The recurrent bouts of sleeping sickness from this infection are a clinical manifestation of the RQ.

**Bibliography**

[1] Agrawal, A. & Lively, C.M. 2002 Infection genetics: gene-for-gene versus matching-alleles models and all points in between. *Evolutionary Ecology Research* **4**, 79-90.

[2] Dybdahl, M.F., Jokela, J., Delph, L.F., Koskella, B. & Lively, C.M. 2008 Hybrid fitness in a locally adapted parasite. *American Naturalist* **172**, 772-782.

[3] Luijckx, P., Ben-Ami, F., Mouton, L., Du Pasquier, L. & Ebert, D. 2011 Cloning of the unculturable parasite *Pasteuria ramosa* and its *Daphnia* host reveals extreme genotype-genotype interactions. *Ecology Letters* **14**, 125-131.

[4] Salvaudon, L., Heraudet, V. & Shykoff, J.A. 2005 Parasite-host fitness trade-offs change with parasite identity: genotype-specific interactions in a plant-pathogen system. *Evolution* **59**, 2518-2524.

[5] Luijckx, P., Fienberg, H., Duneau, D. & Ebert, D. 2013 A matching-allele model explains host resistance to parasites. *Current Biology* **23**, 1085-1088.

[6] Parker, M.A. 1988 Genetic uniformity and disease resistance in a clonal plant. *American Naturalist* **132**, 538-549.

[7] Hedrick, P.W. 1994 Evolutionary genetics of the major histocompatibility complex. *American Naturalist* **143**, 945-964.

[8] Fan, W., Kashara, M., Gutknecht, J., Klein, D., Mayer, W.E., Jonker, M. & Klein, J. 1989 Shared class II MHC polymorphisms between humans and chimpanzees. *Human Immunology* **26**, 107-113.

[9] Figueroa, F., Gunther, E. & Klein, J. 1988 MHC polymorphism pre-dating speciation. *Nature* **335**, 265-267.

[10] Leffler, E.M., Gao, Z., Pfeifer, S., Segurel, L., Auton, A., Venn, O., Bowden, R., Bontrop, R., Wall, J.D., Sella, G., et al. 2013 Multiple Instances of Ancient Balancing Selection Shared Between Humans and Chimpanzees. *Science (New York, N.Y.)* **339**, 1578-1582. (doi:10.1126/science.1234070).

[11] Nielsen, R. 2005 Molecular signatures of natural selection. *Annu Rev Genet* **39**, 197-218.

[12] Obbard, D.J., Jiggins, F.M., Halligan, D.L. & Little, T.J. 2006 Natural selection drives extremely rapid evolution in antiviral RNAi genes. *Current biology : CB* **16**, 580-585.

[13] Obbard, D.J., Welch, J.J., Kim, K.W. & Jiggins, F.M. 2009 Quantifying Adaptive Evolution in the Drosophila Immune System. *PLoS genetics* **5**, e1000698. (doi:10.1371/Journal.Pgen.1000698).

[14] Longdon, B., Wilfert, L., Obbard, D.J. & Jiggins, F.M. 2011 Rhabdoviruses in two species of Drosophila: vertical transmission and a recent sweep. *Genetics* **188**, 141-150.

[15] Metz, E.C. & Palumbi, S.R. 1996 Positive selection for sequence rearrangements generates extensive sequence polymorphism in the gamete recognition protein bindin. *Mol. Biol. Evol.* **13**, 391-406.

[16] Clark, N.L., Gasper, J., Sekino, M., Aquadro, C.F. & Swanson, W.J. 2009 Coevolution of Interacting Fertilization Proteins. *PLoS Genetics* **5**, e1000570.

[17] Frank, S.A. 2000 Sperm competition and female avoidance of polyspermy mediated by sperm-egg biochemistry. *Evolutionary Ecology Research* **2**, 613-625.

[18] Swanson, W.J. & Vacquier, V.D. 1995 Extraordinary divergence and positive Darwinian selection in a fisagenic protein coating the acrosomal process of abalone spermatozoa. *Proc. Natl. Acad. Sci. USA* **92**, 4957-4961.

[19] Palumbi, S.R. 1999 All males are not created equal: Fertility differences depend on gamete recognition polymorphisms in sea urchins. *P Natl Acad Sci USA* **96**, 12632-12637.

[20] Arnqvist, G. & Rowe, L. 2002 Antagonistic coevolution between the sexes in a group of insects. *Nature* **415**, 787-789.

[21] Green, K.K., Kovalev, A., Svensson, E.I. & Gorb, S.N. 2013 Male clasping ability, female polymorphism and sexual conflict: fine-scale elytral morphology as a sexually antagonistic adaptation in female diving beetles. *JOURNAL OF THE ROYAL SOCIETY INTERFACE* **10**, DOI: 10.1098/rsif.2013.0409.

[22] Tregenza, T., Wedell, N. & Chapman, T. 2006 Introduction. Sexual conflict: a new paradigm? *Philosophical Transactions Of The Royal Society B-Biological Sciences* **361**, 229- 234.

[23] Chapman, T., Arnqvist, G., Bangham, J. & Rowe, L. 2003 Sexual conflict. *Trends Ecol. Evol.* **18**, 41-47.

[24] Rowe, L., Cameron, E. & Day, T. 2003 Detecting sexually antagonistic coevolution with population crosses. *P Roy Soc Lond B Bio* **270**, 2009-2016.

[25] Frank, S.A. 2002 *Immunology and evolution of infectious disease*. Princeton (NJ), Princeton University Press.

[26] Rambaut, A., Posada, D., Crandall, K.A. & Holmes, E.C. 2004 The causes and consequences of HIV evolution. *Nat Rev Genet* **5**, 52-61. (doi:10.1038/nrg1246).

[27] Smith, D.J., Lapedes, A.S., de Jong, J.C., Bestebroer, T.M., Osterhaus, A.D. & Fouchier, R.A. 2004 Mapping the antigenic and genetic evolution of influenza virus. *Science (New York, N.Y.)* **305**, 371-376.

[28] Barry, J.D., Hall, J.P.J. & Plenderleith, L. 2012 Genome hyperevolution and the success of a parasite. *Annals of the New York Academy of Sciences* **1267**, 11-17. (doi:10.1111/j.1749-6632.2012.06654.x).
