# Supplementary figures and images for: Running with the Red Queen: the role of biotic conflicts in evolution
Source: Proc Biol Sci. 2014 Dec 22;281(1797):20141382. doi: 10.1098/rspb.2014.1382 (PMC4240979; doi:10.1098/rspb.2014.1382)

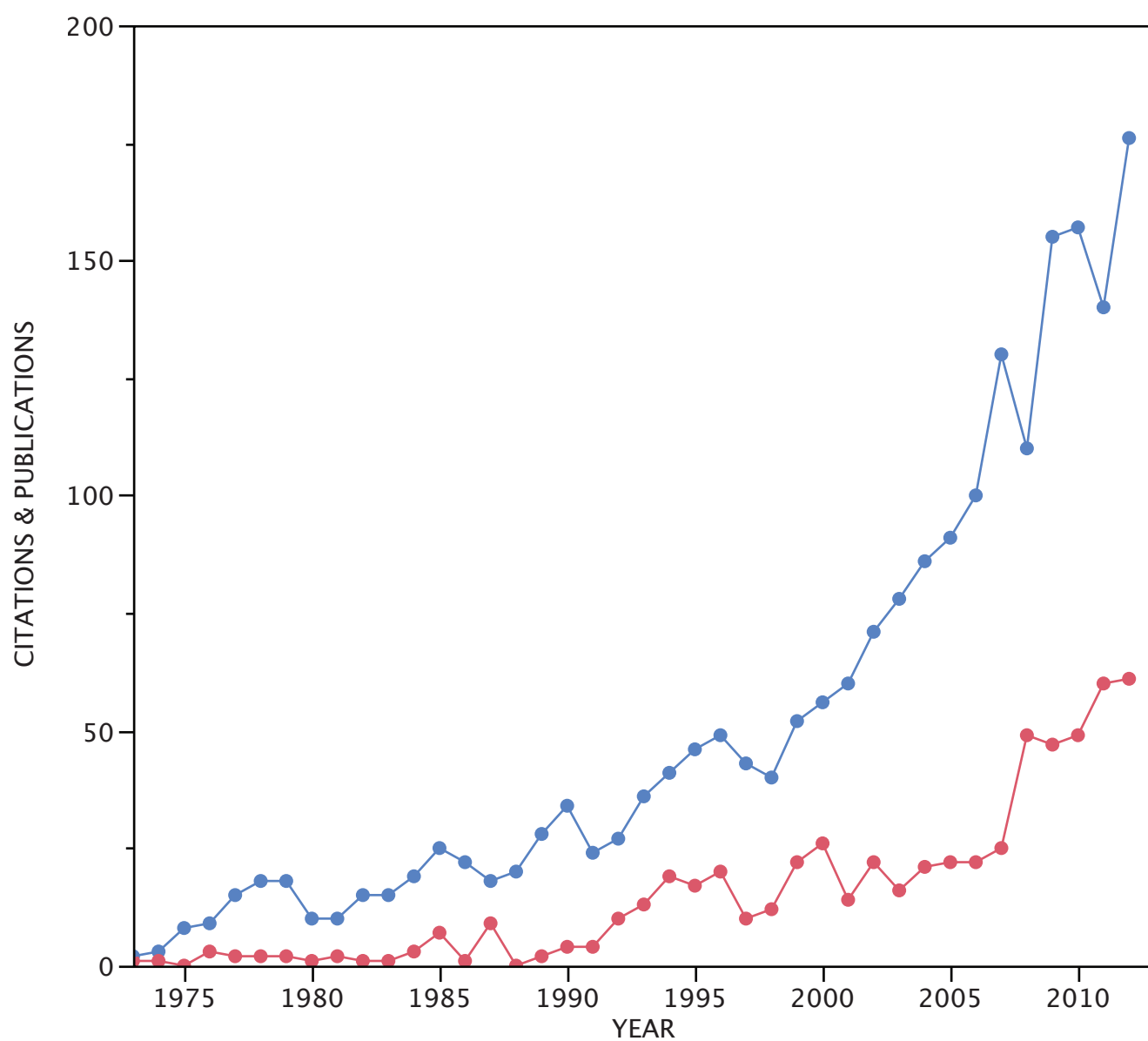

Supplement: Figure S1 [file rspb20141382supp2.pdf]

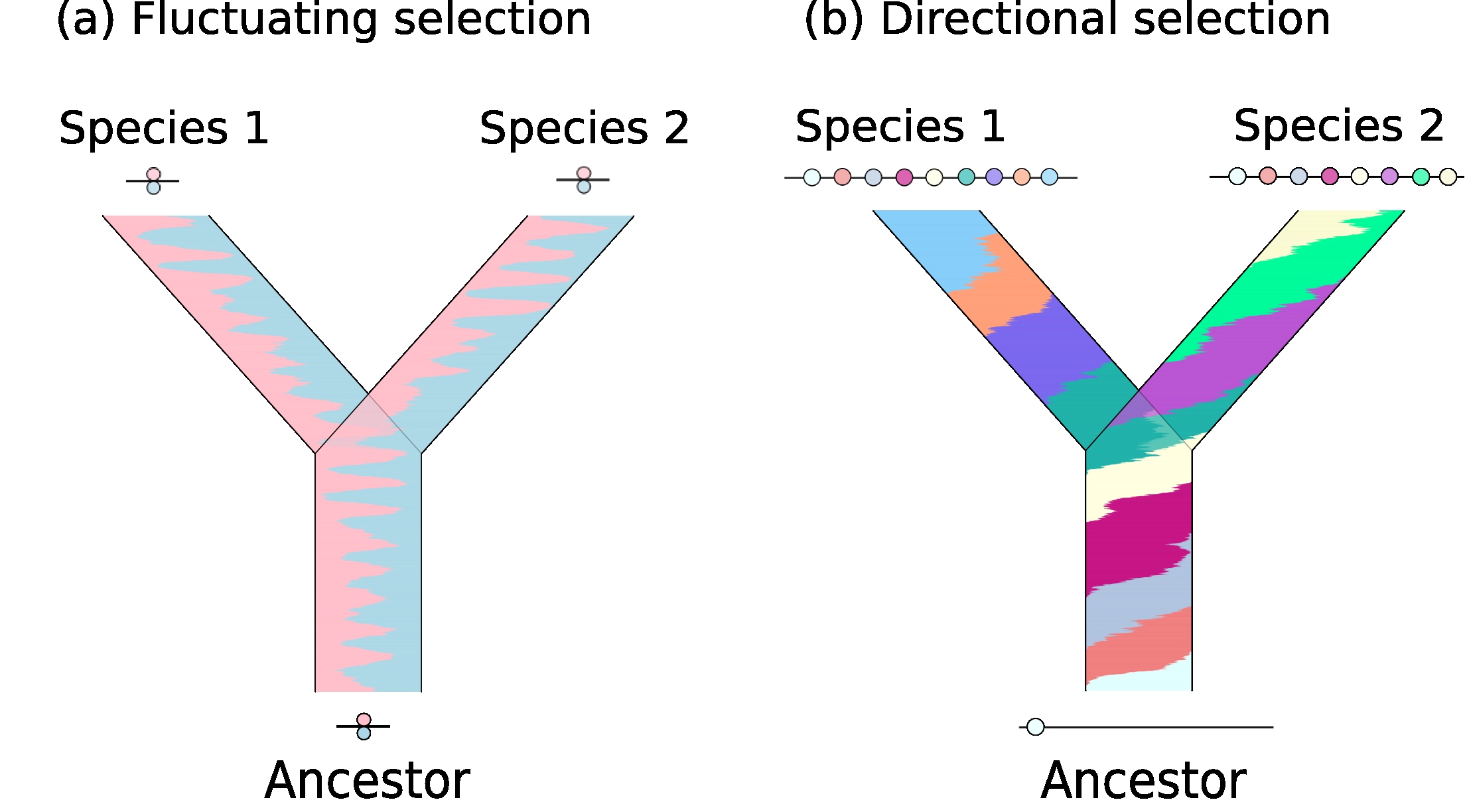

Supplement: Figure S2 [file rspb20141382supp3.jpg]
