# Supplementary material for: Running with the Red Queen: the role of biotic conflicts in evolution
Source: Proc Biol Sci. 2014 Dec 22;281(1797):20141382. doi: 10.1098/rspb.2014.1382 (PMC4240979; doi:10.1098/rspb.2014.1382)
Supplement: Legends for Supplementary Figures [file rspb20141382supp4.docx]

**Legends for Supplementary Figures**

Figure S1: Publication dynamics of the Red Queen hypothesis. Citations (blue line) to "A New Evolutionary Law" were collected from a GoogleScholar search. Publications (red line) were collected from a Web of Science wherein the search term was "Red Queen" in the topic section, years 1973-2012.

Figure S2 (located within Box S1): Genetic diversity resulting from fluctuating (FRQ) and directional selection (ERQ/CRQ). A) Fluctuating selection results in increased genetic diversity within a species, while B) directional selection results in increased diversity between species.
